# The construction and operational models of internet hospitals in China: a hospital-based survey study

**DOI:** 10.1186/s12913-023-09675-2

**Published:** 2023-06-21

**Authors:** Xuejiao Chen, Xinxia Wu, Qihang Zhang, Ran Jing, Weibin Cheng, Junzhang Tian, Changxiao Jin

**Affiliations:** 1 Institute for Healthcare Artificial Intelligence Application, Guangdong Second Provincial General Hospital, Haizhu District, No.466 Xingangzhong Road, Guangzhou, Guangdong, 510317 China; 2 Peking University Third Hospital, Beijing, China; 3 London School of Hygiene and Tropical Medicine, University of London, London, UK; 4 School of Data Science, City University of Hong Kong, Hong Kong S.A.R, China

**Keywords:** Internet hospital, Construction; Establishment model, Hospital-based survey, China

## Abstract

**Background:**

China has empowered and continues to empower internet hospitals, which saw an increase in their development due to the pandemic, to fight against COVID-19. The construction and operational models of internet hospitals can be categorized as self-constructed and self-managed models, self-constructed and enterprise-run models, hospital and enterprise joint-owned models, and hosted by a third-party platform. Despite the growing importance of internet hospitals, there have been few systematic summaries of their construction and operational models. The primary purpose of the study was to understand the construction and operational models of internet hospitals in China.

**Methods:**

Data was collected from 39 internet hospitals and 356 medical staff between September 2020 and April 2021, via internet hospital and hospital staff surveys. T-tests were used to compare the continuous variables, while Chi-square tests were employed to compare the proportions of categorical variables. The self-perception of the internet hospitals’ services was assessed using a 5-point Likert scale on 16 aspects and a root cause analysis was conducted to identify the root causes and influencing factors of current deficiencies experienced by internet hospitals.

**Results:**

Among the 39 internet hospitals, 22 (56.4%) were self-constructed and self-managed. Compared to other models of Internet hospitals, self-constructed and self-managed hospitals had lower percentages of professionals providing online services (*P* = 0.006), numbers of doctors outside of the entity (*P* = 0.006), numbers of online nurses (*P* = 0.004), and the ratio of online nurses to offline doctors (*P* < 0.001). Of the 16 aspects evaluated with regards to the medical staff’s self-perception of the internet hospital services, the highest scores were given for fee transparency, fee rationality, travel cost capital, patience and responsibility, and consultation behaviors. The root causes included five aspects: human, channels, prices, services, and time.

**Conclusions:**

While the self-constructed and self-managed model was found to be the most prevalent form of internet hospital in China, the different models of internet hospitals can have an impact on both the quantity and quality of online healthcare services. This study contributes to the existing literature on internet hospitals' construction and operational models, offering additional policy implications for telemedicine management.

**Supplementary Information:**

The online version contains supplementary material available at 10.1186/s12913-023-09675-2.

## Background

Internet hospital is an innovative approach, similar to telehealth/telemedicine services that combines online and offline access for medical institutions to provide health services to patients [1]. Information technology is used to develop online medical and health services from the hospital to the Internet [2]. Thus, patients who stay at home or visit a local clinic can consult with doctors based in top-level hospitals via a website or smartphone app [3]. Internet hospitals in China are authorized to use internet technology to provide safe and appropriate medical care for common and chronic diseases, such as follow-up treatment or consultation. Doctors can review medical records and prescribe treatment online [4]. As a complement to offline health services, internet hospitals can eliminate geographical and time-related barriers allowing patients to access medical services quickly and conveniently.

The first internet hospital in China was developed in Guangdong Province in 2014 [5]. Between 2014 and 2017, the growth of internet hospitals was relatively slow. At the end of 2018, the Chinese government issued policies to comprehensively regulate internet hospitals, such as diagnosis and treatment, telemedicine, safety and accountability [4]. By January 2019, registered internet hospitals had grown to approximately 130 across 25 provinces [1]. The emergence of COVID-19 has accelerated the development of internet hospitals to supplement suspended in-person healthcare services during the pandemic [6–9]. According to the correspondence by the National Health Commission of China, as of August 2022, more than 1700 internet hospitals have been established nationwide (http://www.nhc.gov.cn/wjw/tia/202208/53b9f3b67ce948e086cb4a67b1e2d8cc.shtml).

The construction and operational models of internet hospitals can be classified as self-constructed and self-managed models, self-constructed and enterprise-run models, hospital and enterprise joint-owned models, and being hosted by a third-party platform according to fund investment, technical forces, and operating management models [10]. Even though much information on the construction and operational models of internet hospitals is yet to be uncovered, Han et al. used the Baidu search engine to define, establish and provide an insight into the development status of internet hospitals up to January 1, 2019 [1]. Han et al. divided internet hospitals into three categories: (1) those initiated by the government, whose primary purpose was to achieve unified standards of regional population health management; (2) those initiated by hospitals, whose primary goal was to expand the scope and intensity of hospital services; and (3) those initiated by enterprises, whose purpose was to connect patients with practicing doctors with the intent of increasing the number of patients and gaining benefits [1]. Xu et al. described the characteristics of internet hospitals from public data and assessed their health service capacity during the COVID-19 pandemic. The results showed that internet hospitals provided various services during the pandemic, such as medical prescriptions, drug delivery, and medical insurance services [8]. Xie et al. reported that many internet hospitals were not yet fully established and faced various challenges, such as insufficient online doctors and unavailability of health insurance coverage [11]. Most internet hospitals are service providers that offer both online healthcare and regular care.

Physicians in internet hospitals may provide a range of medical services, including primary care, specialist, or consultant roles [1]. These physicians may be employed by the internet hospital, contracted to it, or act as independent practitioners. Understanding the nature of physicians’ affiliation within an internet hospital is a crucial factor in determining whether this healthcare service is suitable [3]. This consideration is necessary to ensure that medical services are of the highest quality, meeting the standards of the internet hospital and that physicians have access to the resources and support they need to provide quality care.

As of the first half of 2022, approximately 74% of the Chinese population had access to the Internet, leading to the implementation of a variety of Internet hospitals with promising prospects. However, the sustainability and standardization of these internet hospitals needs to be further established. To assess the development of internet hospitals in China, a survey was conducted with 39 internet hospitals and 356 medical staff to analyze the construction and operational models.

## Methods

### Data collection

#### Internet hospital survey

**Fig. 1:**
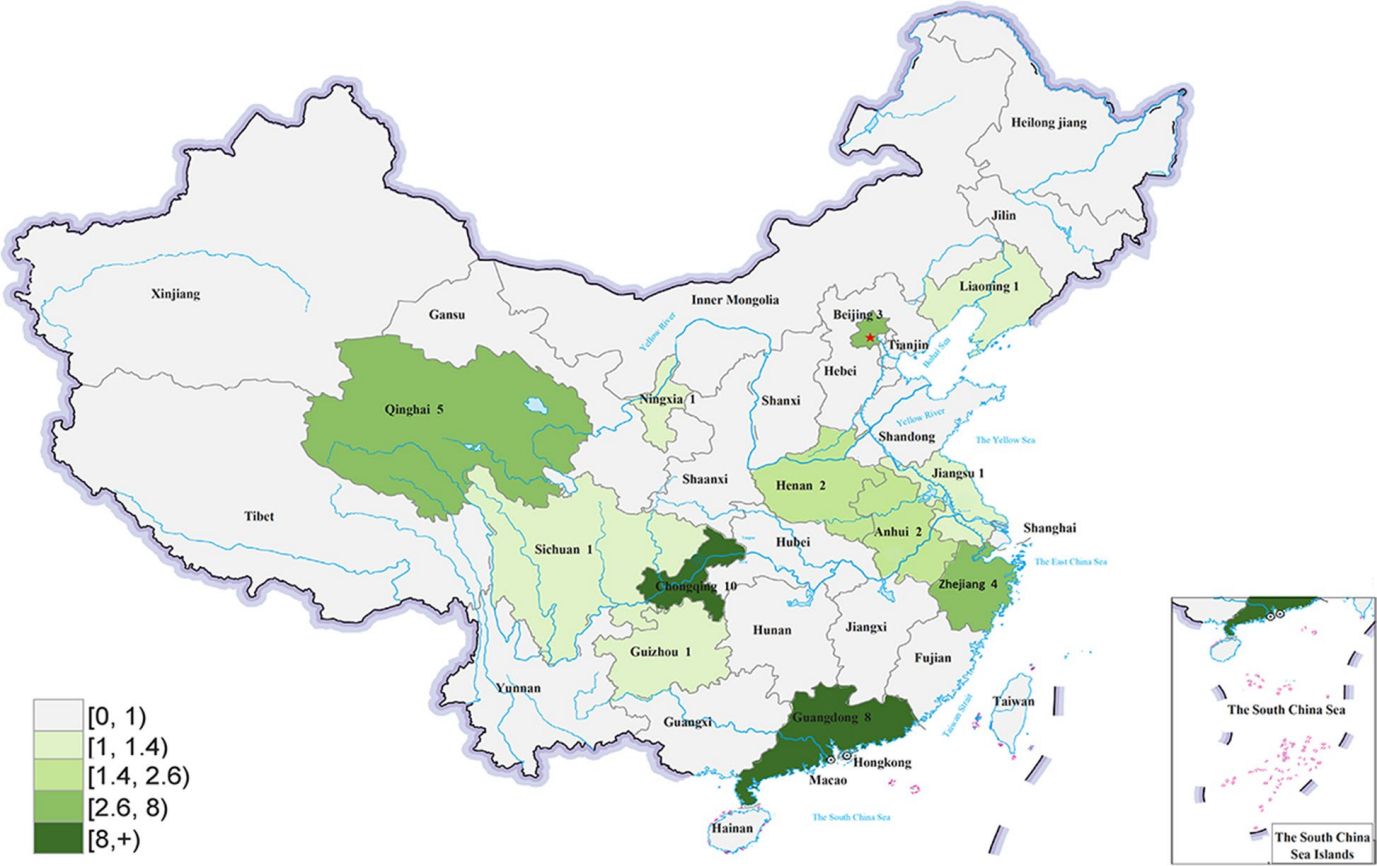
The geographic distribution of the internet hospitals that participated in this study. The numbers represent the number of the internet hospitals surveyed in the provinces (Source: This figure is author’s own work)

This survey was conducted with the support of China’s Internet Hospital Operational and Management Status and Talent Training Program, run by the Chinese National Health Commission. This program comprises 33 network members, which are all types and levels of medical institutes distributed across mainland China (Fig. 1).

There are three types of construction models and four types of operational models. Based on the construction funding source, technical forces, and operating conditions of the internet hospitals, we grouped construction and operational models into four models: self-constructed and self-managed models; self-constructed and enterprise-run models; hospital and enterprise joint-owned models; and those hosted by a third-party platform. The definition of these models was discussed and agreed by the network members through panel discussions [10] (Table 1).

**Table 1:** Types of internet hospital constructions and their operational models

Models	Definitions	Examples
Self-constructed and self-managed models	Medical institutions or enterprises invested in building their own internet hospital and operate on their own	Peking University Third Hospital
Self-constructed and enterprise-run models	Medical institutions invested funding to build their own internet hospital and entrusted a third-party company to manage	Affiliated Cancer Hospital of Chongqing University
Hospital and enterprise joint-owned models	Medical institutions and enterprises joint invested to build the internet hospital and manage the internet hospital together	Eye Ear Nose & Throat Hospital of Zhengzhou University
Internet hospital being hosted by a third-party platform	Medical institutions join in an internet hospital platform which is constructed and managed by a local government or the enterprise platform to provide online health services	Second Hospital Affiliated to Zhengzhou University

Purposive sampling was employed to recruit internet hospitals from across the country. Initially, program members were invited to complete the survey and help to distribute it to hospitals outside of the network. The survey instrument was created by the research team based on the literature [12, 13] and domain knowledge provided by experts within the program network. The questionnaire was pretested in three hospitals (within the research team) to test its acceptability and feasibility. Subsequently, minor adjustments were made regarding the logic and jump after the pretest followed by several rounds of comments and revisions, leading to the development of a well-structured questionnaire approved by the program network members. The survey content for the hospital included information on hospital setup, a quality control plan and an evaluation, and the self-perceptions of internet hospital services. The setup of the internet hospital included an administration plan; information from various healthcare professionals (e.g., doctors, pharmacists, nurses), regulations for quality and safety, an estimated annual operating expenditure, and an estimated annual operating revenue. The self-perception of the internet hospital services was assessed on 17 aspects by using a 5-point Likert scale. Meanwhile, several open-ended questions are included to collect information on the perceived advantages and disadvantages of internet hospitals and attitudes of participants toward providing online medical services, for example: Please list the advantages of internet hospital for the provision of patient services in comparison to services in the entity hospital. An online survey was created using Wenjuanxing (an online survey platform, www.wjx.com) and distributed to the program member network. Each hospital provided information once. All information was collected as of September 2020, and the survey was only administered in Chinese.

#### The hospital staff survey

In addition to collecting data from internet hospitals, we also conducted an individual survey to gain information on the attitudes and perceptions of hospital staff regarding the internet hospitals’ services. The survey’s respondents were health professionals, including general practitioners, specialists, technicians, nurses, administrative staff, who had provided online services during the period of the survey. The development of the hospital staff survey instrument was consistent with the internet hospital survey. The questionnaire consisted of 27 items divided into three parts: 1) demographic information, such as age, degree, and professional title; 2) perceptions of online services provided pre-hospital, in-hospital, and post-hospital visit; and 3) awareness of relevant laws and regulations of the internet hospital. The ratios of administrative staff to clinical staff, online pharmacists to offline doctors, and online nurses to offline doctors were also estimated in order to measure the quantity and quality of internet hospital service delivery. The implementation of the hospital staff survey utilized an online survey platform (Wenjuanxing, www.wjx.com) in Chinese to collect information from September 2020. All participants consented to participate in the online survey and the data used for this report was provided by the program administrations.

### Data analysis

Data analysis was performed using Microsoft Excel 2017 (Microsoft Corp) and the Statistical Package for the Social Sciences (SPSS) software (version 23.0; IBM Corp.). The mean and variance values were calculated for the continuous variables (including the number of administrative staff, doctors, pharmacists, nurses, etc.), and T-tests were employed to compare their values. The categorical variables, such as the characteristics of internet hospitals’ establishment model, regulations for quality and safety, annual operating expenditure, annual operating revenue, and services for patients in the internet hospitals, were described as frequencies and proportions. Chi-square tests were applied to compare the proportions of the categorical variables. The significance level of α was set at 0.05. The self-perceptions of internet hospital services were displayed using a radar chart, and qualitative data (text answers collected by open-ended questions) on improving these services were processed using thematic analysis. Two authors (X.W and W.C) closely examined the data to identify recurring common themes and grouped them into different themes. After the themes were determined, two group discussion sessions were held with 15 specialists from seven internet hospitals, who had a variety of professional backgrounds including hospital management, health informatics, attending physician, nurse, and engineer. The participants had an average of 9.8 years of working experience, 12 (80%) of them held a post-graduate degree, and nine (60%) of them had a senior title. To illustrate the thematic analysis results, two authors (X.C and X.W) developed a root cause analysis diagram.

## Results

### Characteristics of the internet hospitals establishment model

A total of 39 internet hospitals were included in this analysis, of which 30 (76.9%) were general hospitals, nine (23.1%) were specialized hospitals; 31 (79.5%) were tertiary and eight (20.5%) were secondary hospitals; 35 (89.7%) were public hospitals, and four (10.3%) were private hospitals. On terms of regional distribution, 18 (46.2%) were located in eastern China, four (10.3%) in central China, and 17 (43.6%) in western China. With respect to their construction and operational model, the majority of the internet hospitals were self-construction (66.7%) and self-management (56.4%), which were comprised of 22 (56.4%) self-constructed and self-managed internet hospitals (Table 2).

**Table 2:** Characteristics of the internet hospitals’ establishment model

Characteristics	n (%)
**Type of the host hospital**	
General hospital	30 (76.9%)
Specialized hospital	9 (23.1%)
**Level of the host hospital**	
Tertiary	31 (79.5%)
Secondary	8 (20.5%)
**Ownership model**	
Public	35 (89.7%)
Private	4 (10.3%)
**Regions**	
Eastern China	18 (46.2%)
Central China	4 (10.3%)
Western China	17 (43.6%)
**Construction model**^**a**^	
Self-construction	26 (66.7%)
Co-construction	5 (12.8%)
Join to a platform	6 (15.4%)
**Operational model**^**a**^	
Self-management	22 (56.4%)
Third-party management	6 (15.4%)
Jointly-run management	5 (12.8%)
Enterprise-run management	4 (10.3%)
**Internet hospital models**^**a**^	
Self-constructed and self-managed	22 (56.4%)
Self-constructed and enterprise-run	4 (10.3%)
Hospital and enterprise joint-owned	5 (12.8%)
Being hosted by a third-party platform	6 (15.4%)

^a^Two internet hospitals were under development

### Characteristics of the internet hospitals’ setup

The characteristics of the internet hospital setup information are summarized in Table 3. There are two types of variables in Table 3: countable variables and numeric variables. Countable variables are presented as counts and percentages [n (%)], while numeric variables are presented as mean and standard deviation [Mean (SD)]. For countable variables, column 2 should be equal to the sum of values in columns 3 and 4. However, for numeric variables, column 2 may not be equal to the sum of columns 3 and 4. A comparison between self-constructed and self-managed hospitals and other models of hospitals revealed statistical differences in the proportion of professionals providing online services (*P* = 0.006), the number of doctors outside the entity (*P* = 0.006), the number of online nurses (*P* = 0.004), the ratio of online nurses to offline doctors (*P* < 0.001), and whether it related to the provincial regulatory platform (*P* = 0.033). The self-constructed and self-managed hospitals had a lower proportion of physicians providing online services, a smaller number of doctors outside the entity, a smaller number of online nurses, and a lower ratio of online nurses to offline doctors than other models of hospitals. These four ratios represent the personnel structure of internet hospitals. The relatively high ratios of these indicators in other models of internet hospitals suggest that more investment has been made in their construction and operation, which may affect the quantity and quality of internet hospital services. Furthermore, self-constructed and self-managed hospitals were more likely to be connected to the provincial regulatory platform than other models of hospitals (Table 3).

**Table 3:** Characteristics of the internet hospitals’ setup^a^

	**Total**	**By models**		
**Characteristics**	**n (%) / Mean (sd)**	**Self-constructed and self-managed** **n (%) / Mean (sd)**	**Other models**^**b**^ **n (%) / Mean (sd)**	***P***^**†**^
**Administration department of internet hospital**				
Number of medical institutions with independent internet hospital management departments	18 (46.2%)	11 (28.2%)	7 (17.9%)	0.748
Number of administrative staff	9 (5)	6 (4)	10(6)	0.133
The ratio of administrative staff to clinical staff	0.05 (0.02)	0.03 (0.01)	0.06 (0.03)	**0.001**
**Online doctors**				
Number of online doctors	172 (255)	182 (295)	153 (170)	0.745
Number of doctors in the entity	336 (372)	373 (434)	270 (222)	0.413
Number of doctors in outside of entity	7 (19)	4 (12)	31 (46)	**0.006**
Proportions of professionals providing online services	29.2 (26.0)	21.4 (21.2)	44.9 (28.3)	**0.006**
**Online pharmacists**				
Number of online pharmacists	14 (23)	13 (26)	16 (12)	0.706
Number of pharmacists in the entity	30 (50)	30 (59)	33 (26)	0.873
Number of pharmacists in outside of entity	1 (2)	0	1 (2)	-
The ratio of online pharmacists to offline doctors	0.2 (0.3)	0.2 (0.3)	0.3 (0.3)	0.094
**Online nurses**				
Number of online nurses	21 (49)	10 (42)	64 (53)	**0.004**
Number of nurses in the entity	309 (420)	248 (444)	432 (349)	0.201
Number of nurses in outside of entity	3 (11)	0	3 (11)	-
The ratio of online nurses to offline doctors	< 0.1 (< 0.1)	< 0.01(< 0.1)	0.2 (0.1)	**0.001**
**Regulations for quality and safety**				
Whether it is interconnected with HIS\LIS\PACS data of offline hospitals	29 (74.4%)	19 (48.7%)	10 (25.6%)	0.071
Whether it is connected with the provincial regulatory platform	28 (71.8%)	19 (48.7%)	9 (23.1%)	0.033
Whether the diagnoses and prescriptions are electronically signed by the doctors	34 (87.2%)	20 (51.3%)	14 (35.9%)	0.636
Whether all patients have electronic medical records	34 (87.2%)	19 (48.7%)	15 (38.5%)	1.000
Are all prescriptions reviewed by pharmacists	34 (87.2%)	20 (51.3%)	14 (35.9%)	0.636
**Annual operating expenditure**				
Less than 500 000 Yuan	23 (59.0%)	14 (35.9%)	9 (23.1%)	
500 000 to 1 million	10 (25.6%)	6 (15.4%)	4 (10.3%)	0.593
Above 1 million	6 (15.4%)	2 (5.1%)	4 (10.3%)	
**Annual operating revenue**				
Less than 500 000 Yuan	23 (60.0%)	15 (38.5%)	8 (20.5%)	
500 000 to 1 million	5 (12.8%)	1 (2.6%)	4 (10.3%)	0.214
Above 1 million	11 (28.2%)	6 (15.4%)	5 (12.8%)	

^†^The t test was used to compare values of continuous variables. The Chi square test was used to compare proportions of categorical variables

^a^Information of the status of internet hospital was collected as of September 2020. Numbers of people were rounded to the nearest whole number. Countable variables are presented as counts and percentages [n (%)]; and numeric variables are presented as mean and standard deviation [Mean (SD)]

^b^Other models included self-constructed and enterprise-run model, hospital and enterprise joint owned model, and hosted by a third-party platform

### Direct-to-patient services available in the internet hospitals

Online consultation and follow-up visits were the two items with the highest proportion of available direct-to-patient services in internet hospitals. Electronic prescriptions, drug delivery, and health education followed in terms of availability. Health education showed a significant statistical difference between self-constructed and self-managed hospitals and other models of hospitals (*P* = 0.008). Self-constructed and self-managed hospitals had a higher percentage of health education. Additionally, drug delivery exhibited a marginal statistical difference between self-constructed and self-managed hospitals and other models of hospitals (*P* = 0.057), as shown in Table 4.

**Table 4:** Available services for patients in the internet hospitals^a^

	**Total**	**By models**		***P***
**Classification**	**n (%)**	Self-constructed and self-managed **n (%)**	**Other models**^**b**^ **n (%)**	
Online consultation	29 (74.4%)	17 (43.6%)	12 (30.8%)	0.721
Follow-up visit	24 (61.5%)	15 (38.5%)	9 (23.1%)	0.508
Electronic prescription	21 (53.8%)	14 (35.9%)	7 (17.9%)	0.206
Drug delivery	21 (53.8%)	15 (38.5%)	6 (15.4%)	0.057
Prescribe examination	6 (15.4%)	5 (12.8%)	1 (2.6%)	0.206
Make an appointment for examination	5 (12.8%)	4 (10.3%)	1 (2.6%)	0.363
Referral to the physical hospital	4 (10.3%)	3 (7.7%)	1 (2.6%)	0.618
Health education	17 (43.6%)	14 (35.9%)	3 (7.7%)	0.008

^a^Information of the status of internet hospital was collected as of September 2020

^b^Other models refer to self-constructed and enterprise-run model, hospital and enterprise joint owned model, and hosted by a third-party platform

### Self-scoring of internet hospital services

**Fig. 2:**
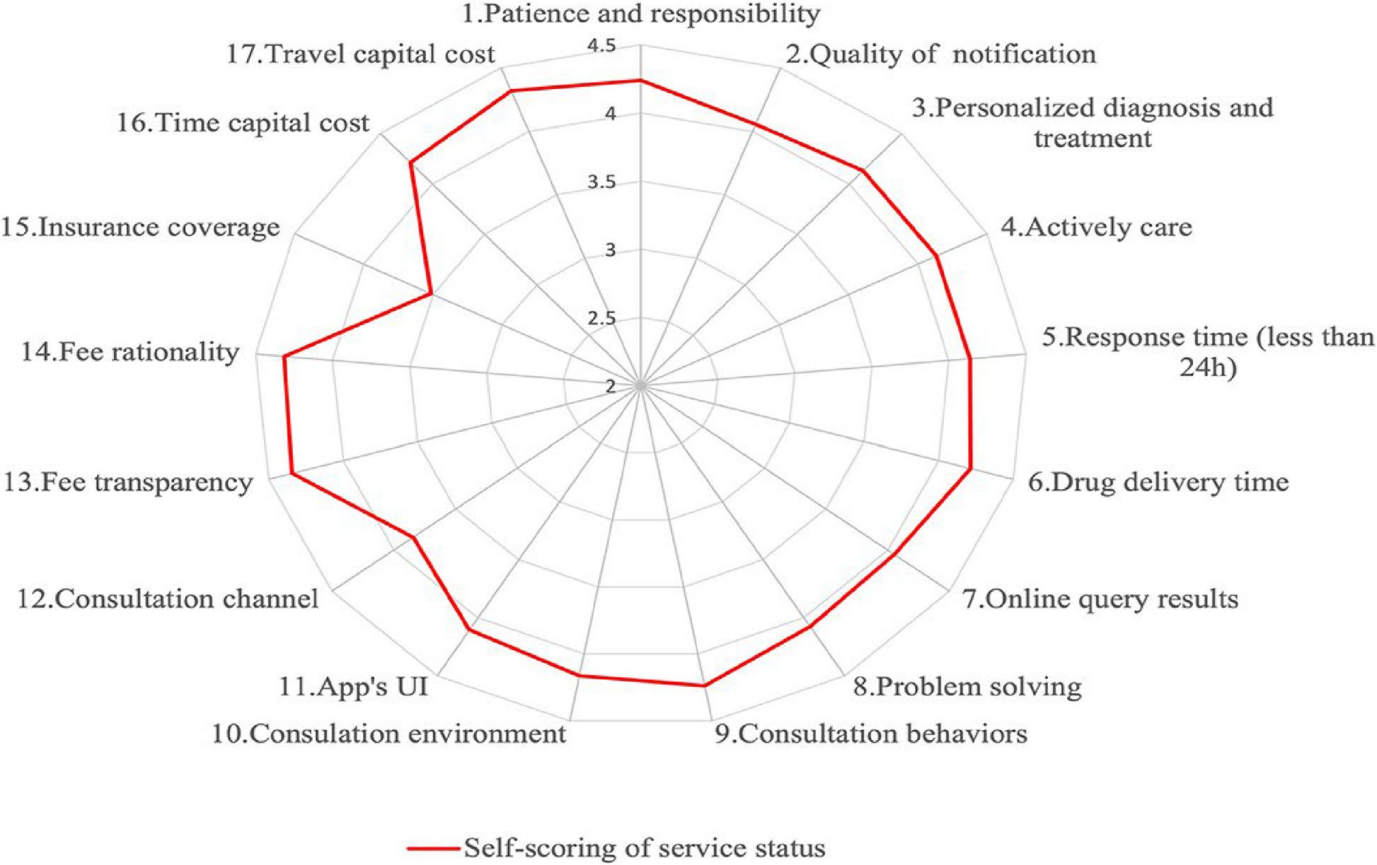
The hospital staff’s self-perception of the internet hospital services

The average score that individuals gave to internet hospital services was 4.12 ± 0.19. As seen in Fig. 2, the top five evaluation items ranked by their average score were fee transparency, fee rationality, travel cost capital, patience and responsibility, and consultation behaviors. The items that scored the lowest were insurance coverage, consultation channel, quality of notification, online query results, and problem solving.

### The root causes for improving internet hospitals’ services

**Fig. 3:**
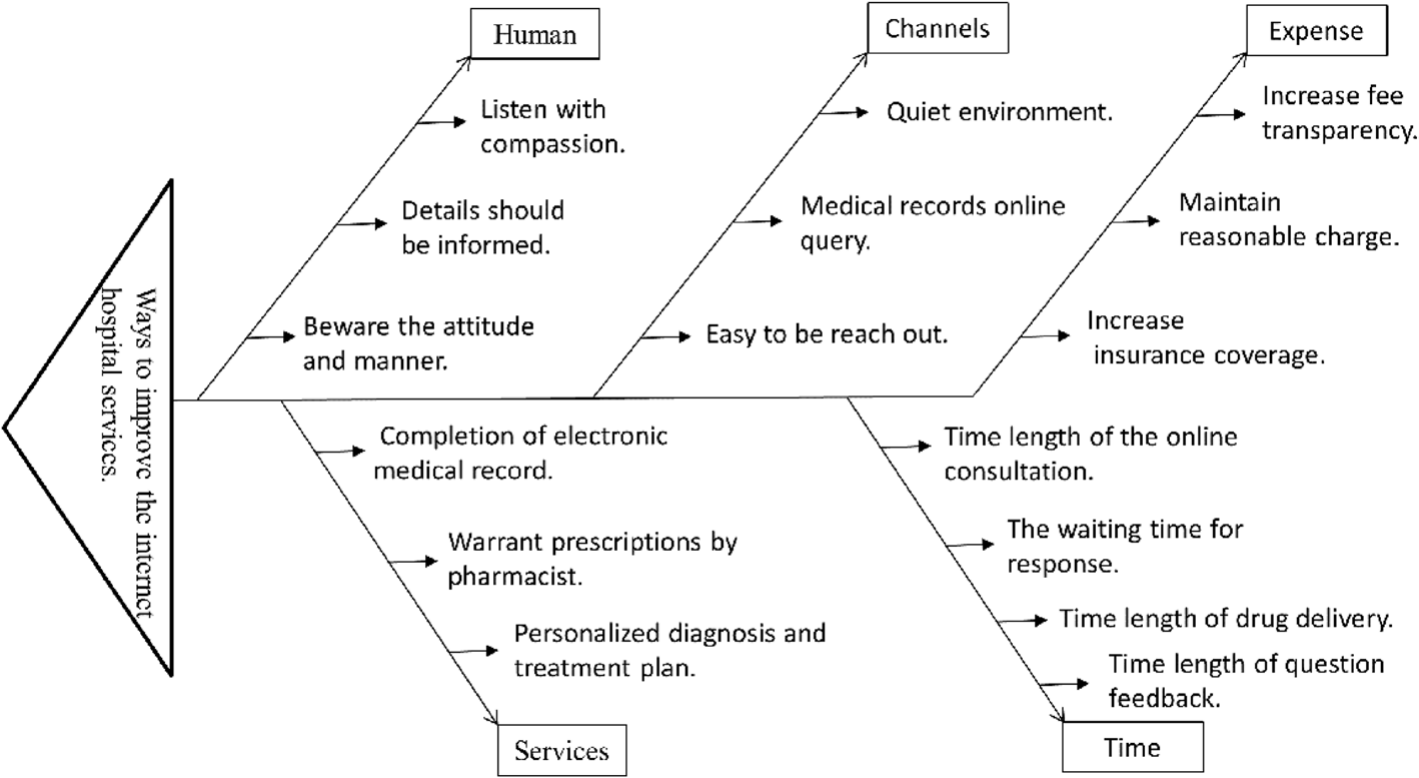
The root causes analysis diagram for the internet hospital services

Analysis of the service conditions of internet hospitals indicated five major dimensions or factors that can affect the quality of hospital services: human, channels, prices, services, and time. These five dimensions have 16 related elements, as depicted in Fig. 3. Root cause analysis (fishbone diagram) was then used to determine the underlying causes of any deficiencies found in the current internet hospital services (Fig. 3). Definition of terms used in Fig. 3 were provided in Additional file 1 appendix.

## Discussion

### Principal findings

The results showed that the self-constructed and self-managed model was the major type among the four construction and operational models of internet hospitals in China. It is worth noting that the proportion of professionals providing online services, the number of doctors outside the entity, number of online nurses, and the ratio of online nurses to offline doctors of self-constructed and the self-operated internet hospitals were significantly lower than those of other models. The reasons for this may be attributed to differences in doctors’ technical levels, hospital departments, and workloads between the self-constructed and self-managed models and those of other models of internet hospitals [14]. Furthermore, different types of internet hospitals have different stakeholders with different supporting content and responsibilities.

There was no difference between the self-constructed and self-managed models and other models on the availability of direct-to-patient services in internet hospitals, except for health education. The self-constructed and self-managed internet hospitals invested funds, built their own technical forces, and operated services independently [15]. Other investigators have reported that perceived behavioral control and perceived severity of disease were the most influential determinants of patients’ intention to use online inquiry services provided by the internet hospitals [16].

The professional technology of medical services determines the indispensability of the doctors. With the support of information technology, internet hospitals promoted the balance of medical resources and improved the convenience and efficiency of medical services. Doctors, as medical service providers, will only use the platform if they endorse internet hospitals and make the most of it. To gain an understanding the medical staff’s attitude towards internet hospitals, it is necessary to investigate them. We found that health professionals perceived that internet hospital’s services could work well in terms of fee transparency, fee rationality, travel cost capital, patience and responsibility, and consultation behaviors. Meanwhile, online consultation is essential in increasing patients’ trust and satisfaction. Doctors should be aware of patients’ intention to visit in person following an online consultation. Good communication is beneficial in fostering strong doctor-patient relationships [17, 18].

### Implications and suggestions for future efforts

First, the construction of an internet hospital requires coordination and cooperation between multiple departments. Therefore, it is recommended to establish an independent internet hospital management department, which is more efficient for mobilization and coordination. If an independent management department is not currently established, it is suggested that the medical manager, who is responsible for the main management, take on the management role and assume the corresponding responsibilities. Meanwhile, it is recommended that a service management system for internet hospitals be established, considering three aspects: quality and safety, process standardization, and information security [19]. Of these, patient safety and the risk of treatment errors are particularly important. Medical errors indicate weaknesses in complex systems. Therefore, to reduce the risk of harm, greater emphasis should be placed on identifying risk factors, ensuring effective communication with patients, raising situation awareness, strengthening management and standardized operation, and ensuring quality [20].

Second, the establishment of a connection between internet hospitals and provincial internet medical service supervision platforms is one the prerequisites for the application and approval of qualifications for internet hospital in China. Additionally, it is also essential to implement third-level information security protection in the information system. Writing medical records and prescriptions is the requirement of the core diagnosis and treatment component of internet hospitals [21]. To meet the requirements for qualification access, medical or third-party institutions should continue to expand other information businesses.

Third, to strengthen information security management, hospitals should continuously improve the level of information; effectively standardize the use, collection, development, and ownership of data; strictly verify the identity and access rights; steadily improve the level of data encryption; and preferably ensure the integrity, confidentiality, and availability of information [22]. Moreover, cloud computing and security mechanisms can be used in conjunction to enhance the security and operability of information systems [23, 24].

Fourth, internet hospitals should manage the entire cycle of health, supplementing the relatively limited business functions of offline physical medical institutions while providing convenient services to patients. Functional businesses should be continuously expanded in accordance with demands. Strengthening the professional knowledge of health professionals and introducing multidisciplinary talent in information technology is also important.

### Limitations

Several limitations should be considered when interpreting the results. First, the number of internet hospitals has been increasing during the COVID-19 pandemic, meaning this study may not adequately reflect the wider subject of internet hospitals in China. Thus, the possibility that the participants in this study may or may not be representative of all facilities in China. Nevertheless, the construction and operational models of these hospitals are relatively stable, since the regulations of internet hospital establishments have not yet been modified. Second, this study only surveyed the medical staff of these hospitals to evaluate their service capacity, ignoring the patient’s perspective. It is essential to also examine the satisfaction of medical services provided by the internet hospitals and analyze their implementation in the current situation [16, 25].

### Comparison with prior work

Few studies have reported on the construction and operational models of internet hospitals. Cui et al. focused on the implementation, application, and influencing factors of telemedicine [15]. Jiang et al. studied the characteristics of online consultations and inquiries through China's largest online medical platform [26]. Using a social exchange theory perspective, Ren et al. examined the effect of doctors' internet-based service quality on their economic returns during the COVID-19 social restrictions [27]. Another study discussed the policy interventions, development trends, and service innovations of internet hospitals in China, based on a documentary analysis and qualitative interview studies [28]. In Arabic countries, Alsayed’s study suggested that a large number of patients need expanding telemedicine and pharmaceutical care services and were willing to pay for them during COVID-19 pandemic [29]. In Europe, a national online survey among doctors, nurses, and other medical professionals in Germany found that the perception of telemedicine during the current COVID-19 crisis was generally favorable throughout all professional groups, indicating the potential for telemedicine to evolve from model implementations to a telemedical structure when technical and regulatory challenges can be addressed [30].

## Conclusions

Internet hospitals are revolutionizing the provision of medical service provision through the use of telemedicine technology. This has enabled tailored medical interventions for various types of patients, making such services indispensable for not only COVID-19 but also for other health services. The findings of this study revealed that the self-constructed and self-managed model is the most common form of internet hospital model in China. However, the various models of internet hospitals could impact the quantity and quality of online healthcare services. Longitudinal studies are needed to further examine the construction and operational models of internet hospitals. Nonetheless, this study can provide useful information for policymaking and help in guiding further internet hospital practice in China.

## Supplementary Information

**Additional file 1. Appendix**

## Data Availability

The dataset of this study will be made available upon reasonable request of the corresponding author.

## Abbreviations

COVID-19: Coronavirus disease 2019
HIS: Hospital information system
LIS: Laboratory information system
PACS: Picture archiving communication system
App: Application
UI: User interface
