# Supplementary material for: The construction and operational models of internet hospitals in China: a hospital-based survey study
Source: BMC Health Serv Res. 2023 Jun 21;23:669. doi: 10.1186/s12913-023-09675-2 (PMC10283228; doi:10.1186/s12913-023-09675-2)
Supplement: Supplementary file 1 — Additional file 1. Appendix [file 12913_2023_9675_MOESM1_ESM.docx]

**Appendix**

# The Construction and Operational Models of Internet Hospitals in China: A Hospital-Based Survey Study

Definitions of terms used in Figure 3.

**Human factors**

**Listen with compassion** refers to the practice of actively and empathetically listening to a patient's concerns and needs when providing online medical support or assistance. This involves acknowledging the patient's emotions, showing understanding and empathy towards their medical condition or situation, and providing medical advice or support in a caring and non-judgmental manner.

**Detail should be informed** refers to the principle that all relevant details and information about a patient's medical condition should be clearly communicated and shared with the patient when providing online medical support or assistance. This includes providing all necessary information about the medical service being provided, such as its features, limitations, and potential risks or benefits, as well as any relevant policies or guidelines that may impact the patient's use of the service.

**Beware the attitude and manner** refers to the importance of maintaining a positive, professional, and caring attitude and manner when interacting with patients who are seeking medical advice or support online. This includes being courteous, respectful, and attentive to the patient's needs and concerns, as well as being patient and understanding when addressing any medical issues or challenges that may arise.

**Channel factors**

**Quiet environment** refers to a physical or virtual environment that is free from noise, distractions, and interruptions when providing medical support or assistance online. This includes creating a space that is conducive to concentration, focus, and reflection, and that allows the medical service provider to fully engage with the patient's medical condition and needs.

**Medical records online query** refers to the ability for individuals to access and search their own medical records through an online platform. This can include viewing information such as medical diagnoses, treatment plans, medication lists, test results, and other relevant health information that has been recorded by healthcare providers.

**Easy to be reach out** refers to the accessibility of hospitals or healthcare providers via various communication channels, such as phone, email, or online chat. It means that patients can easily contact the hospital or healthcare provider to get medical advice, schedule appointments, or ask questions about their health. This is important for providing high-quality healthcare services and ensuring that patients feel supported and cared for.

**Expense factors**

**Increase fee transparency** refers to the practice of providing clear and accessible information about the fees and costs associated with using a particular medical service online. This can include providing detailed information about the cost of the service, any additional fees or charges, and any discounts or promotions that may be available.

**Maintain reasonable charge** refers to the obligation of hospitals or healthcare providers to offer their online medical services at a price that is fair and reasonable, taking into account the nature of the service, the costs of providing the service, and the prevailing market conditions.

**Increase insurance coverage** refers to the expansion of insurance plans to cover online medical services provided by hospitals or healthcare providers. This means that patients can use their insurance to pay for virtual consultations, telemedicine visits, and other online medical services offered by internet hospital services.

**Services factors**

**Completion of electronic medical records** refers to the process of creating and maintaining digital records of patients' health information, diagnosis, treatments, and other medical information. This information is stored electronically and can be accessed by healthcare providers through a secure online platform.

**Warrant prescriptions by pharmacist** refers to the practice of having a pharmacist review and verify prescriptions for medications that have been issued online by a healthcare provider. This can include verifying the accuracy of the prescription, checking for potential drug interactions, and ensuring that the medication is appropriate for the patient's medical condition and needs.

**Personalized diagnosis and treatment plan** refers to the practice of tailoring medical diagnosis and therapy to the individual needs and characteristics of each patient when providing medical support or advice online. This can include using patient-specific data, such as medical history, genetic information, and lifestyle factors, to inform medical diagnosis and therapy recommendations.

**Time factors**

**Time length of online consultation** refers to the duration of a virtual consultation between a patient and a healthcare provider conducted through an online platform. This can range from a few minutes for a quick check-up or follow-up visit to an extended appointment for a more complex medical issue. The time length of online consultation is an important consideration for internet hospital services as it impacts the quality and effectiveness of the medical care provided. Healthcare providers need to allocate sufficient time for each virtual consultation to ensure that they can address the patient's medical concerns, provide accurate diagnosis and treatment recommendations, and answer any questions the patient may have.

**The waiting time for response** refers to the amount of time that elapses between a patient's request for medical assistance or information and the healthcare provider's response. This can refer to the time it takes for a patient to receive a reply to an email or online message, the time it takes for a healthcare provider to connect with the patient for a virtual consultation, or the time it takes for a patient to receive medical test results or other important information. The waiting time for response is an important aspect of internet hospital services as it impacts the patient's experience and satisfaction with the service.

**Time length of drug delivery** refers to the time taken for the ordered drugs to be dispensed and shipped to the patient after an e-prescription is received and filled by the online pharmacy or medical service. It indicates how quickly drugs are made available to patients after an online consultation and prescription.

**Time length of question feedback** indicates how quickly the patient's queries or posted questions are responded to by the doctors or healthcare professionals. It is a metric of the responsiveness of the internet-based hospital service and their timeliness in providing feedback or answers to patients. A shorter 'time length of question feedback' is typically seen as a positive indicator of quality of service.
